# A New Variational Bayesian-Based Kalman Filter with Unknown Time-Varying Measurement Loss Probability and Non-Stationary Heavy-Tailed Measurement Noise

**DOI:** 10.3390/e23101351

**Published:** 2021-10-16

**Authors:** Chenghao Shan, Weidong Zhou, Yefeng Yang, Hanyu Shan

**Affiliations:** 1 Department of Intelligent Systems Science and Engineering, Harbin Engineering University, Harbin 150001, China; shanchenghao123@hrbeu.edu.cn; 2 Center for Control Theory and Guidance Technology, Harbin Institute of Technology, Harbin 150001, China; 18B904013@stu.hit.edu.cn; 3 Department of Information and Communication Engineering, Harbin Engineering University, Harbin 150001, China; shy@hrbeu.edu.cn

**Keywords:** variational Bayesian, Kalman filter, measurement loss probability, mixture distribution, non-stationary heavy-tailed measurement noise

## Abstract

In this paper, a new variational Bayesian-based Kalman filter (KF) is presented to solve the filtering problem for a linear system with unknown time-varying measurement loss probability (UTVMLP) and non-stationary heavy-tailed measurement noise (NSHTMN). Firstly, the NSHTMN was modelled as a Gaussian-Student’s *t*-mixture distribution via employing a Bernoulli random variable (BM). Secondly, by utilizing another Bernoulli random variable (BL), the form of the likelihood function consisting of two mixture distributions was converted from a weight sum to an exponential product and a new hierarchical Gaussian state-space model was therefore established. Finally, the system state vector, BM, BL, the intermediate random variables, the mixing probability, and the UTVMLP were jointly inferred by employing the variational Bayesian technique. Simulation results revealed that in the scenario of NSHTMN, the proposed filter had a better performance than current algorithms and further improved the estimation accuracy of UTVMLP.

## 1. Introduction

Under the minimal mean square error criteria, the KF is the optimal estimator for the linear Gaussian state-space model [1,2]. KF has been widely employed in a variety of applications [3,4,5]. Unfortunately, in many practical applications, when the sensor produces intermittent faults, the actual measurement of the sensors may not be accurately represented by the KF measurement model [6,7]. If the random measurement loss occurs, the measurement of the sensors contains only pure noise. In this situation, the estimation accuracy of a typical KF will drop significantly or even diverge. Various filtering methods have been developed to address the measurement loss filtering issue, such as the intermittent KF (IKF) [8,9]. However, IKF has an important assumption: the measurement loss probability is known. In practical applications, the measurement loss probability is usually unknown and the IKF is no longer applicable in this case [7].

In order to address the filtering issues of the unknown measurement loss probability of the linear system, the first Bayesian Kalman filter and the second Bayesian Kalman filter were designed by estimating the process and posterior distribution of the measurement loss, respectively [10]. The above two filters, however, are no longer valid if the unknown measurement loss probability is time-varying. Recently, the variational Bayesian-based adaptive KF (VBAKF) was derived for a linear system with unknown time-varying measurement loss probability (UTVMLP) and both the system vector and UTVMLP are jointly estimated by introducing the variational Bayesian technique [7]. Additionally, VBAKF shows excellent performance in the context of white Gaussian measurement noise with known statistical characteristics. Unfortunately, in realistic engineering applications, measurement outliers may occur at various periods due to environmental changes and unreliable sensors, resulting in NSHTMN, i.e., when the system runs healthily, the measurement noise is the Gaussian-distributed, and when the time-varying measurement outliers erode the system, the measurement noise is heavy-tail-distributed [11,12]. In the scenario of NSHTMN, the estimation accuracy of VBAKF will drop sharply.

Recently, some mixture distribution-based algorithms have been presented to address NSHTMN, such as the Gaussian-Student’s *t*-mixture distribution-based KF (GSTKF) [13,14]. However, the filtering problem with UTVMLP and NSHTMN cannot directly solved by employing a mixture distribution, that is, under the scenario of UTVMLP and NSHTMN, the current likelihood function is a weighted sum of double-mixture distributions, which is an unclosed and unconjugated distribution that makes the Bayesian inference difficult to employ directly.

In this paper, a new variational Bayesian-based KF is presented to settle the filtering issue for linear discrete-time systems with UTVMLP and NSHTMN. Firstly, the Gaussian-Student’s *t*-mixture distribution with BM is employed to model the NSHTMN. Secondly, the form of the likelihood function is converted to an exponential product and constructs a new hierarchical Gaussian state-space model by utilizing BL. Thirdly, the variational Bayesian method is introduced to simultaneously estimate the system state vector, BM, BL, the intermediate random variables, the mixing probability, and the UTVMLP. Finally, a numerical simulation experiment reveals that the proposed filter has better estimation accuracy but is more time-consuming than existing filtering algorithms in the scenarios of NSHTMN and UTVMLP.

The contributions of this paper are as follows:

(a) By employing a Bernoulli-distributed variable, the NSHTMN is modelled as a Gaussian-Student’s *t*-mixture distribution;
(b) The measurement likelihood function is converted from the weight sum of two mixture distributions to an exponential product and a new hierarchical Gaussian state-space model is therefore derived;
(c) The system state vector, UTVMLP, and the unknown variables are simultaneously estimated by utilizing the variational Bayesian technique;
(d) Numerical simulation results indicate that the proposed filter has better performance than that of existing algorithms in the scenarios of NSHTMN and UTVMLP

## 2. Problem Formulation

Consider the linear stochastic system with the following state and measurement equations:

(1) $$x_{t}=F_{t-1}x_{t-1}+e_{t-1}$$

(2) $$y_{t}=b_{t}H_{t}x_{t}+g_{t}$$

where $x_{t}\in {\mathbb{R}}^{m}$ denotes the system state vector; $F_{t-1}\in {\mathbb{R}}^{m\times m}$ denotes the state transition matrix; $e_{t}\in {\mathbb{R}}^{m}$ represents the Gaussian-distributed white process noise vector with a zero mean value and covariance matrix $Q_{t}$; $y_{t}\in {\mathbb{R}}^{n}$ represents the measurement vector; $H_{t}\in {\mathbb{R}}^{n\times m}$ is the measurement matrix; $g_{t}\in {\mathbb{R}}^{n}$ is the white NSHTMN vector; and t represents the index of discrete time. The phenomenon of measurement loss is described by introducing the identically distributed and mutually uncorrelated measurement loss defined as the Bernoulli random variable (BL) $b_{t}$, which is expressed by the following equations.

(3) $$p\left(b_{t}=1\right)=E\left[b_{t}\right]=\gamma_{t}$$

(4) $$p\left(b_{t}=0\right)=1-E\left[b_{t}\right]=1-\gamma_{t}$$

where $\gamma_{t}\in \left[0,1\right]$ denotes the time-varying measurement loss probability. Note that the value of $\gamma_{t}$ is unknown in this paper. The initial Gaussian-distributed system state vector $x_{0}$ is the random vector with mean ${\hat{x}}_{0|0}=0$ and covariance matrix $P_{0|0}$. Additionally, it is assumed that the initial system state vector $x_{0}$, the noise vectors $e_{t-1}$ and $g_{t}$, and the Bernoulli random variable $b_{t}$ are mutually independent.

It can be seen from Equations (1)–(4) that the ideal measurement was received by the sensor when $b_{t}=0$ and the measurement loss with UTVMLP occurred when $b_{t}=1$. Meanwhile, the measurement noise is NSHTMN due to measurement outliers, that is, when the system runs healthily, the measurement noise is Gaussian-distributed, and when measurement outliers erode the system, the measurement noise is heavy-tail-distributed. The NSHTMN and UTVMLP can result in estimation errors or even in filtering divergence. Therefore, a new variational Bayesian-based Kalman filter with NSHTMN and UTVMLP will be proposed.

## 3. Proposed Variational Bayesian-Based Kalman Filter

In this section, a new variational Bayesian-based Kalman filter is proposed to address the filtering issue for a linear system with NSHTMN and UTVMLP. Firstly, the Gaussian-Student’s *t*-mixture distribution is utilized to model the NSHTMN and the hierarchical form is derived. Secondly, by converting the measurement likelihood function into an exponential multiplication, a new hierarchical Gaussian state-space model is established. Thirdly, by using the variational Bayesian method, the system state and unknown variables are simultaneously estimated. Finally, the required mathematical expectations are given.

### 3.1. Gaussian-Student’s t-Mixture Distribution

The NSHTMN vector can be modeled as the Gaussian-Student’s *t*-mixture distribution by employing another mixing-defined Bernoulli random variable (BM), $\zeta_{t}$, and the probability density function (PDF), $p\left(g_{t}\right)$ is given as

(5) $$p\left(g_{t}\right)=\overset{1}{\underset{\zeta_{t}=0}{\sum }}\int {\left[N\left(g_{t};0,R_{t}\right)\right]}^{\zeta_{t}}{\left[\mathrm{ST}\left(g_{t};0,R_{t},\mu \right)\right]}^{\left(1-\zeta_{t}\right)}p\left(\zeta_{t}|\varphi_{t}\right)p\left(\varphi_{t}\right)d\varphi_{t}\;\zeta_{t}\in \left\{0,1\right\}$$

where N(x;0,Σ) represents the Gaussian PDF with a zero mean vector and covariance matrix Σ, and ST(x;0,Υ,ω) represents the student’s *t*-PDF with a zero mean vector, covariance matrix Υ, and degree of freedom (dof) parameter ω. $R_{t}$ represents the covariance matrix of the nominal measurement noise. The PDF of the mixing probability $\varphi_{t}$ and the probability mass function (PMF) of $\zeta_{t}$ are defined as follows, respectively.

(6) $$p\left(\varphi_{t}\right)=\mathrm{Be}\left(\varphi_{t};h_{0},1-h_{0}\right)$$

(7) $$p\left(\zeta_{t}|\varphi_{t}\right)=\varphi_{t}{}^{\zeta_{t}}{\left(1-\varphi_{t}\right)}^{\left(1-\zeta_{t}\right)}$$

where Be(x;σ,κ) represents the Beta PDF with shape parameters σ and κ.

Due to the hierarchical properties of the student’s *t*-distribution, Equation (5) can be rewritten as such:

(8) $$p\left(g_{t}\right)=\overset{1}{\underset{\zeta_{t}=0}{\sum }}\int_{0}^{+\infty }\int {\left[N\left(g_{t};0,R_{t}\right)\right]}^{\zeta_{t}}{\left[N\left(g_{t};0,R_{t}/\beta_{t}\right)\right]}^{\left(1-\zeta_{t}\right)}p\left(\beta_{t}\right)p\left(\zeta_{t}|\varphi_{t}\right)p\left(\varphi_{t}\right)d\varphi_{t}d\beta_{t}$$

(9) $$p\left(\beta_{t}\right)=G\left(\beta_{t},\frac{\mu }{2},\frac{\mu }{2}\right)$$

where G(x,a,b) represents the Gamma PDF with shape parameter a and rate parameter b, and $\beta_{t}$ represents the intermediate random variable.

### 3.2. New Hierarchical Gaussian State-Space Model (HGSSM)

According to Equations (2)–(4), the measurement likelihood PDF is derived as Based on Equation (2), the following equation can be obtained.

(10) $$\begin{array}{ll} p\left(y_{t}|x_{t},\gamma_{t}\right) & =\overset{1}{\underset{b_{t}=0}{\sum }}p\left(y_{t},b_{t}|x_{t},\gamma_{t}\right)\\ & =p\left(y_{t}|x_{t},b_{t}=1\right)p\left(b_{t}=1\right)+p\left(y_{t}|x_{t},b_{t}=0\right)p\left(b_{t}=0\right)\\ & =\left(1-\gamma_{t}\right)p\left(y_{t}|x_{t},b_{t}=1\right)+\gamma_{t}p\left(y_{t}|x_{t},b_{t}=0\right) \end{array}$$

(11) $$p\left(y_{t}|x_{t},b_{t}=1\right)=p_{g_{t}}\left(y_{t}-H_{t}x_{t}\right)$$

(12) $$p\left(y_{t}|x_{t},b_{t}=0\right)=p_{g_{t}}\left(y_{t}\right)$$

where $p_{g_{t}}\left(\cdot \right)$ represents the measurement noise PD. Substituting Equations (11) and (12) in Equation (10) results in

(13) $$p\left(y_{t}|x_{t},\gamma_{t}\right)=\left(1-\gamma_{t}\right)p_{g_{t}}\left(y_{t}-H_{t}x_{t}\right)+\gamma_{t}p_{g_{t}}\left(y_{t}\right)$$

**Remark** **1.** The measurement likelihood PDF in Equation (13) is an unclosed and unconjugated weighted sum form, and it is impossible to infer the system state vector and unknown parameters directly by utilizing the variational Bayesian. The weighted sum will then be converted into an exponential multiplication form to address this problem.

The PMF of BL $b_{t}$ is given as

(14) $$p\left(b_{t}|\gamma_{t}\right)={\left(1-\gamma_{t}\right)}^{b_{t}}\gamma_{t}{}^{\left(1-b_{t}\right)}$$

Exploiting Equations (13) and (14), the measurement likelihood PDF is reformulated as

(15) $$\begin{array}{ll} p\left(y_{t}|x_{t},\gamma_{t}\right) & =\overset{1}{\underset{b_{t}=0}{\sum }}p\left(y_{t}|x_{t},b_{t}\right)p\left(b_{t}|\gamma_{t}\right)\\ & =\overset{1}{\underset{b_{t}=0}{\sum }}\left[{\left(1-\gamma_{t}\right)}^{b_{t}}{\left[p_{g_{t}}\left(y_{t}-H_{t}x_{t}\right)\right]}^{b_{t}}\gamma_{t}{}^{\left(1-b_{t}\right)}{\left[p_{g_{t}}\left(y_{t}\right)\right]}^{\left(1-b_{t}\right)}\right]\\ & =\overset{1}{\underset{b_{t}=0}{\sum }}\left[{\left[p_{g_{t}}\left(y_{t}-H_{t}x_{t}\right)\right]}^{b_{t}}{\left[p_{g_{t}}\left(y_{t}\right)\right]}^{\left(1-b_{t}\right)}\right]p\left(b_{t}|\gamma_{t}\right) \end{array}$$

According Equation (15), the exponential multiplication-formed likelihood PDF $p\left(y_{t}|x_{t},b_{t}\right)$ is given as follows.

(16) $$p\left(y_{t}|x_{t},b_{t}\right)={\left[p_{g_{t}}\left(y_{t}-H_{t}x_{t}\right)\right]}^{b_{t}}{\left[p_{g_{t}}\left(y_{t}\right)\right]}^{\left(1-b_{t}\right)}$$

**Remark** **2.** The variational Bayesian method must select the suitable conjugate-prior distributions for unknown variables. Therefore, the appropriate prior PDFs to construct a new HGSSM are selected.

The one-step predicted PDF $p\left(x_{t}|y_{1:t-1}\right)$ of system state vector $x_{t}$ is assumed as being Gaussian distributed as follows.

(17) $$p\left(x_{t}|y_{1:t-1}\right)=N\left(x_{t};{\hat{x}}_{t|t-1},P_{t|t-1}\right)$$

where ${\hat{x}}_{t|t-1}$ represents the mean vector and $P_{t|t-1}$ represents the covariance matrix. Both ${\hat{x}}_{t|t-1}$ and $P_{t|t-1}$ can be updated by the typical Kalman filter, which is given as

(18) $${\hat{x}}_{t|t-1}=F_{t-1}{\hat{x}}_{t-1|t-1}$$

(19) $$P_{t|t-1}=H_{t-1}P_{t-1|t-1}H_{t-1}^{T}+Q_{t-1}$$

In employing Equations (8), (9) and (16), the conditional likelihood PDF $p\left(y_{t}|x_{t},b_{t},\beta ,\zeta_{t}\right)$ is derived as

(20) $$\begin{array}{ll} p\left(y_{t}|x_{t},b_{t},\beta_{t},\zeta_{t}\right) & ={\left[N{\left(y_{t};H_{t}x_{t},R_{t}\right)}^{\zeta_{t}}N{\left(y_{t};H_{t}x_{t},R_{t}/\beta_{t}\right)}^{\left(1-\zeta_{t}\right)}\right]}^{b_{t}}\\ & \times {\left[N{\left(y_{t};0,R_{t}\right)}^{\zeta_{t}}N{\left(y_{t};0,R_{t}/\beta_{t}\right)}^{\left(1-\zeta_{t}\right)}\right]}^{\left(1-b_{t}\right)}\\ & =N{\left(y_{t};H_{t}x_{t},R_{t}\right)}^{\zeta_{t}b_{t}}N{\left(y_{t};H_{t}x_{t},R_{t}/\beta_{t}\right)}^{\left(1-\zeta_{t}\right)b_{t}}\\ & \times N{\left(y_{t};0,R_{t}\right)}^{\zeta_{t}\left(1-b_{t}\right)}N{\left(y_{t};0,R_{t}/\beta_{t}\right)}^{\left(1-\zeta_{t}\right)\left(1-b_{t}\right)} \end{array}$$

It can be seen from Equations (6)–(9), (13) and (20) that the measurement vector $y_{t}$ depends on system state vector $x_{t}$, intermediate random variable $\beta_{t}$, BM $\zeta_{t}$, BL $b_{t}$, mixing probability $\varphi_{t}$, and measurement loss probability $\gamma_{t}$. The following joint-prior PDF must be calculated, i.e.,

(21) $$\begin{array}{c} p\left(\Xi |y_{1:t-1}\right)=p\left(x_{t}|y_{1:t-1}\right)p\left(\beta_{t}\right)p\left(b_{t}|\gamma_{t}\right)p\left(\zeta_{t}|\varphi_{t}\right)p\left(\varphi_{t}\right)p\left(\gamma_{t}|y_{1:t-1}\right),\\ \Xi ≜\left\{x_{t},\beta_{t},b_{t},\zeta_{t},\gamma_{t},\varphi \right\} \end{array}$$

where the definitions of $p\left(\gamma_{t}\right)$, $p\left(\zeta_{t}|\varphi \right)$, p(β), $p\left(b_{t}|\gamma_{t}\right)$, and $p\left(x_{t}|y_{1:t-1}\right)$ are given in Equations (6), (7), (9), (14) and (17), respectively. Additionally, $p\left(\gamma_{t}|y_{1:t-1}\right)$ denotes the prior PDF of the time-varying measurement loss probability, which can be assumed as the following Beta distribution.

(22) $$\begin{array}{ll} p\left(\gamma_{t}|y_{1:t-1}\right) & =\mathrm{Be}\left(\gamma_{t};{\hat{\eta }}_{t|t-1},{\hat{\delta }}_{t|t-1}\right)\\ & =\int p\left(\gamma_{t-1}|y_{1:t-1}\right)p\left(\gamma_{t}|\gamma_{t-1}\right)d\gamma_{t-1}\left(\mathrm{Bayes}’\;\mathrm{theorem}\right) \end{array}$$

where the shape parameters ${\hat{\eta }}_{t|t-1}$ and ${\hat{\delta }}_{t|t-1}$ can be calculated by introducing the forgetting factor ρ∈(0,1] as follows.

(23) $${\hat{\eta }}_{t|t-1}=\rho {\hat{\eta }}_{t-1|t-1}$$

(24) $${\hat{\delta }}_{t|t-1}=\rho {\hat{\delta }}_{t-1|t-1}$$

where ${\hat{\eta }}_{t-1|t-1}$ and ${\hat{\delta }}_{t-1|t-1}$ represent posterior shape parameters.

The proposed new HGSSM is comprised of Equations (14) and (17)–(24). System state vector $x_{t}$, intermediate random variable $\beta_{t}$, BM $\zeta_{t}$, BL $b_{t}$, mixing probability $\varphi_{t}$, and measurement loss probability $\gamma_{t}$ will be simultaneously estimated by utilizing the variational Bayesian method.

### 3.3. Variational Bayesian Approximation of the Joint Posterior PDFs

Aiming at the estimation of the unknown variables of the new HGSSM, the joint posterior PDF $p\left(\Xi |y_{t}\right)$ with $\Xi ≜\left\{x_{t},\beta_{t},b_{t},\zeta_{t},\varphi_{t},\gamma_{t}\right\}$ is required to be solved. However, the analytical solution of $p\left(\Xi |y_{t}\right)$ is not accessible. The variational Bayesian approach is therefore employed to determine an approximate PDF for $p\left(\Xi |y_{t}\right)$ and to compute an approximate solution [15,16,17], i.e.,

(25) $$p\left(\Xi |y_{1:t}\right)\approx q_{a}\left(x_{t}\right)q_{a}\left(\beta_{t}\right)q_{a}\left(b_{t}\right)q_{a}\left(\zeta_{t}\right)q_{a}\left(\varphi_{t}\right)q_{a}\left(\gamma_{t}\right)$$

where θ represents an arbitrary element of Ξ and $q_{a}\left(\theta \right)$ denotes the approximate PDF or PMF. By minimizing the Kullback–Leibler divergence (KLD) between $p\left(\Xi |y_{1:t}\right)$ and $q_{a}\left(x_{t}\right)q_{a}\left(\beta_{t}\right)q_{a}\left(b_{t}\right)q_{a}\left(\zeta_{t}\right)q_{a}\left(\varphi_{t}\right)q_{a}\left(\gamma_{t}\right)$, $q_{a}\left(\theta \right)$ can be obtained as follows.

(26) $$\begin{array}{c} \left\{q_{a}\left(x_{t}\right),q_{a}\left(\beta_{t}\right),q_{a}\left(b_{t}\right),q_{a}\left(\zeta_{t}\right),q_{a}\left(\varphi_{t}\right),q_{a}\left(\gamma_{t}\right)\right\}=\mathrm{argminKLD}\\ \left(q_{a}\left(x_{t}\right)q_{a}\left(\beta_{t}\right)q_{a}\left(b_{t}\right)q_{a}\left(\zeta_{t}\right)q_{a}\left(\varphi_{t}\right)q_{a}\left(\gamma_{t}\right)||p\left(\Xi |y_{1:t}\right)\right) \end{array}$$

(27) $$\mathrm{KLD}\left(q_{a}\left(A\right)||p\left(A\right)\right)≜\int q_{a}\left(A\right)\log\frac{q_{a}\left(A\right)}{p\left(A\right)}dA$$

where $\mathrm{KLD}\left(q_{a}\left(A\right)||p\left(A\right)\right)$ represents the KLD between $q_{a}\left(A\right)$ and p(A), and the optimal solution of Equation (26) can be calculated via the following formula [15,17].

(28) $$\log q_{a}\left(\theta \right)=E_{\Xi^{-\theta }}\left[\log p\left(\Xi ,y_{1:t}\right)\right]+c_{\theta }$$

where E(θ) denotes the mathematical expectation operation, $\Xi^{-\theta }$ signifies a grouping of all the components in Ξ apart from θ, and the constant with regard to θ is denoted by $c_{\theta }$. Additionally, the fixed-point iteration technique is utilized to derive the approximate formation of $q_{a}\left(\theta \right)$ due to the fact that estimated parameters are mutually coupled.

The joint PDF $p\left(\Xi ,y_{1:t}\right)$ in Equation (26) can be derived as

(29) $$\begin{array}{ll} p\left(\Xi ,y_{1:t}\right) & =N{\left(y_{t};H_{t}x_{t},R_{t}\right)}^{\zeta_{t}b_{t}}N{\left(y_{t};H_{t}x_{t},R_{t}/\beta_{t}\right)}^{\left(1-\zeta_{t}\right)b_{t}}\\ & \times N{\left(y_{t};0,R_{t}\right)}^{\zeta_{t}\left(1-b_{t}\right)}N{\left(y_{t};0,R_{t}/\beta_{t}\right)}^{\left(1-\zeta_{t}\right)\left(1-b_{t}\right)}N\left(x_{t};{\hat{x}}_{t|t-1},P_{t|t-1}\right)\\ & \times G\left(\beta_{t},\frac{\mu_{t}}{2},\frac{\mu_{t}}{2}\right)\varphi_{t}{}^{\zeta_{t}}{\left(1-\varphi_{t}\right)}^{\left(1-\zeta_{t}\right)}{\left(1-\gamma_{t}\right)}^{b_{t}}\gamma_{t}{}^{\left(1-b_{t}\right)}\\ & \times \mathrm{Be}\left(\varphi_{t};h_{0},1-h_{0}\right)\mathrm{Be}\left(\gamma_{t};{\hat{\eta }}_{t|t-1},{\hat{\delta }}_{t|t-1}\right)p\left(y_{1:t-1}\right) \end{array}$$

**Proposition** **1.** Let $\theta =x_{t}$ and by using Equation (29) in (28), $q_{a}^{\left(s+1\right)}\left(x_{t}\right)$ can be updated as Gaussian, i.e.,

(30) $$q_{a}^{\left(s+1\right)}\left(x_{t}\right)=N\left(x_{t};{\hat{x}}_{t|t}^{\left(s+1\right)},P_{t|t}^{\left(s+1\right)}\right)$$

where $q_{a}^{\left(s+1\right)}\left(\cdot \right)$ represents the approximate PDF in the (s+1)th iteration, while the mean vector ${\hat{x}}_{t|t}^{\left(s+1\right)}$ and the covariance matrix $P_{t|t}^{\left(s+1\right)}$ are assumed to be updated in accordance with the traditional Kalman filter as follows.

(31) $${\hat{x}}_{t|t}^{\left(s+1\right)}={\hat{x}}_{t|t-1}+K_{t}^{\left(s+1\right)}\left(y_{t}-H_{t}{\hat{x}}_{t|t-1}\right)$$

(32) $$P_{t|t}^{\left(s+1\right)}={\hat{P}}_{t|t-1}^{\left(s+1\right)}-K_{t}^{\left(s+1\right)}H_{t}{\hat{P}}_{t|t-1}^{\left(s+1\right)}$$

(33) $$K_{t}^{\left(s+1\right)}={\hat{P}}_{t|t-1}^{\left(s+1\right)}H_{t}^{T}{\left(H_{t}{\hat{P}}_{t|t-1}^{\left(s+1\right)}H_{t}^{T}+{\tilde{R}}_{t}^{\left(s+1\right)}\right)}^{-1}$$

where $K_{t}^{\left(s+1\right)}$ represents the Kalman gain matrix. The modified measurement noise covariance matrix at (s+1)th iteration ${\tilde{R}}_{t}^{\left(s+1\right)}$ is formulated as

(34) $${\tilde{R}}_{t}^{\left(s+1\right)}=\frac{R_{t}}{E^{\left(s\right)}\left[\zeta_{t}\right]E^{\left(s\right)}\left[b_{t}\right]+E^{\left(s\right)}\left[1-\zeta_{t}\right]E^{\left(s\right)}\left[b_{t}\right]E^{\left(s\right)}\left[\beta_{t}\right]}$$

where $E^{\left(s\right)}\left[\cdot \right]$ represents the mathematical expectation of variables in the sth iteration.

**Proof:** see Appendix A.

**Proposition** **2.** Let $\theta =\beta_{t}$ and by using Equation (29) in Equation (28), $q_{a}^{\left(s+1\right)}\left(\beta_{t}\right)$ can be updated as Gamma, i.e.,

(35) $$q_{a}^{\left(s+1\right)}\left(\beta_{t}\right)=G\left(\beta_{t};\pi_{t}^{\left(s+1\right)},\nu_{t}^{\left(s+1\right)}\right)$$

where the shape parameter $\pi_{t}^{\left(s+1\right)}$ and rate parameter $\nu_{t}^{\left(s+1\right)}$ are formulated as

(36) $$\pi_{t}^{\left(s+1\right)}=0.5\left(nE^{\left(s+1\right)}\left[1-\zeta_{t}\right]+\mu_{t}\right)$$

(37) $$\nu_{t}^{\left(s+1\right)}=0.5\left[\mathrm{tr}\left(G_{t}^{\left(s+1\right)}R_{t}^{-1}\right)+\mu_{t}\right]$$

where n represents the dimension of the measurement vector, tr(·) represents the trace operation, and $G_{t}^{\left(s+1\right)}$ is defined as

(38) $$\begin{array}{ll} G_{t}^{\left(s+1\right)} & =E^{\left(s\right)}\left[1-\zeta_{t}\right]\{E^{\left(s\right)}\left[b_{t}\right]\left[H_{t}P_{t|t}^{\left(s+1\right)}H_{t}^{T}+\left(y_{t}-H_{t}{\hat{x}}_{t|t}^{\left(s+1\right)}\right){\left(y_{t}-H_{t}{\hat{x}}_{t|t}^{\left(s+1\right)}\right)}^{T}\right]\\ & +E^{\left(s\right)}\left[1-\zeta_{t}\right]E^{\left(s\right)}\left[1-b_{t}\right]y_{t}{\left(y_{t}\right)}^{T}\} \end{array}$$

**Proof:** see Appendix B.

**Proposition** **3.** Let $\theta =b_{t}$ and by using Equation (29) in Equation (28), $q_{a}^{\left(s+1\right)}\left(b_{t}\right)$ can be updated as the Bernoulli distribution. The posterior probabilities $p\left(b_{t}=1\right)$ and $p\left(b_{t}=0\right)$ of BL $b_{t}$ are given as

(39) $$p^{\left(s+1\right)}\left(b_{t}=1\right){∆}^{\left(s+1\right)}\exp\left\{E^{\left(s\right)}\left[\log\left(1-\gamma_{t}\right)\right]-0.5\mathrm{tr}\left(C_{t}^{\left(s+1\right)}R_{t}^{-1}\right)+0.5nE^{\left(s\right)}\left[1-\zeta_{t}\right]E^{\left(s\right)}\left[\log\left(\beta_{t}\right)\right]\right\}$$

(40) $$p^{\left(s+1\right)}\left(b_{t}=0\right)={∆}^{\left(s+1\right)}\exp\left\{E^{\left(s\right)}\left[\log\left(\gamma_{t}\right)\right]-0.5\mathrm{tr}\left(D_{t}^{\left(s+1\right)}R_{t}^{-1}\right)+0.5nE^{\left(s\right)}\left[1-\zeta_{t}\right]E^{\left(s\right)}\left[\log\left(\beta_{t}\right)\right]\right\}$$

where ${∆}^{\left(s+1\right)}$ represents the normalizing constant and the parameters $C_{t}^{\left(s+1\right)}$ and $D_{t}^{\left(s+1\right)}$ are, respectively, defined as

(41) $$C_{t}^{\left(s+1\right)}=\left\{E^{\left(s\right)}\left[\zeta_{t}\right]+E^{\left(s\right)}\left[\beta_{t}\right]E^{\left(s\right)}\left[1-\zeta_{t}\right]\right\}\left[H_{t}P_{t|t}^{\left(s+1\right)}H_{t}^{T}+\left(y_{t}-H_{t}{\hat{x}}_{t|t}^{\left(s+1\right)}\right){\left(y_{t}-H_{t}{\hat{x}}_{t|t}^{\left(s+1\right)}\right)}^{T}\right]$$

(42) $$D_{t}^{\left(s+1\right)}=\left\{E^{\left(s\right)}\left[\zeta_{t}\right]+E^{\left(s\right)}\left[\beta_{t}\right]E^{\left(s\right)}\left[1-\zeta_{t}\right]\right\}y_{t}y_{t}^{T}$$

**Proof:** see Appendix C.

**Proposition** **4.** Let $\theta =\zeta_{t}$ and by using Equation (29) in (28), $q_{a}^{\left(s+1\right)}\left(\zeta_{t}\right)$ can be also updated as the Bernoulli distribution. The posterior probabilities $p\left(\zeta_{t}=1\right)$ and $p\left(\zeta_{t}=0\right)$ of BM $\zeta_{t}$ are given as

(43) $$p^{\left(s+1\right)}\left(\zeta_{t}=1\right)=\nabla^{\left(s+1\right)}\exp\left\{E^{\left(s\right)}\left[\log\left(\varphi_{t}\right)\right]-0.5\mathrm{tr}\left(V_{t}^{\left(s+1\right)}R_{t}^{-1}\right)\right\}$$

(44) $$p^{\left(s+1\right)}\left(\zeta_{t}=0\right)=\nabla^{\left(s+1\right)}\exp\left\{E^{\left(s\right)}\left[\log\left(1-\varphi_{t}\right)\right]-0.5\mathrm{tr}\left(W_{t}^{\left(s+1\right)}R_{t}^{-1}\right)+0.5nE^{\left(s\right)}\left[\log\left(\beta_{t}\right)\right]\right\}$$

where $\nabla^{\left(s+1\right)}$ also represents the normalizing constant and the definitions of parameters $V_{t}^{\left(s+1\right)}$ and $W_{t}^{\left(s+1\right)}$ are, respectively, given as

(45) $$\begin{array}{ll} V_{t}^{\left(s+1\right)} & =E^{\left(s\right)}\left[b_{t}\right]\left[H_{t}P_{t|t}^{\left(s+1\right)}H_{t}^{T}+\left(y_{t}-H_{t}{\hat{x}}_{t|t}^{\left(s+1\right)}\right){\left(y_{t}-H_{t}{\hat{x}}_{t|t}^{\left(s+1\right)}\right)}^{T}\right]\\ & +E^{\left(s\right)}\left[1-b_{t}\right]y_{t}y_{t}^{T} \end{array}$$

(46) $$\begin{array}{ll} W_{t}^{\left(s+1\right)} & =E^{\left(s\right)}\left[b_{t}\right]E^{\left(s\right)}\left[\beta_{t}\right]\left[H_{t}P_{t|t}^{\left(s+1\right)}H_{t}^{T}+\left(y_{t}-H_{t}{\hat{x}}_{t|t}^{\left(s+1\right)}\right){\left(y_{t}-H_{t}{\hat{x}}_{t|t}^{\left(s+1\right)}\right)}^{T}\right]\\ & +E^{\left(s\right)}\left[1-b_{t}\right]E^{\left(s\right)}\left[\beta_{t}\right]y_{t}y_{t}^{T} \end{array}$$

**Proof:** see Appendix D.

**Proposition** **5.** Let $\theta =\varphi_{t}$ and by using Equation (29) in (28), $q_{a}^{\left(s+1\right)}\left(\varphi_{t}\right)$ can be updated as the Beta distribution, i.e.,

(47) $$q_{a}^{\left(s+1\right)}\left(\varphi_{t}\right)=\mathrm{Be}\left(\varphi_{t};h_{t}^{\left(s+1\right)},d_{t}^{\left(s+1\right)}\right)$$

where the shape parameters $h_{t}^{\left(s+1\right)}$ and $d_{t}^{\left(s+1\right)}$ are formulated as

(48) $$h_{t}^{\left(s+1\right)}=E^{\left(s+1\right)}\left[\zeta_{t}\right]+h_{0}$$

(49) $$d_{t}^{\left(s+1\right)}=E^{\left(s+1\right)}\left[1-\zeta_{t}\right]+1-h_{0}$$

**Proof:** see Appendix E.

**Proposition** **6.** Let $\theta =\gamma_{t}$ and by using Equation (29) in Equation (28), $q_{a}^{\left(s+1\right)}\left(\gamma_{t}\right)$ can be also updated as the Beta distribution, i.e.,

(50) $$q_{a}^{\left(s+1\right)}\left(\gamma_{t}\right)=\mathrm{Be}\left(\gamma_{t};{\hat{\eta }}_{t}^{\left(s+1\right)},{\hat{\delta }}_{t}^{\left(s+1\right)}\right)$$

where the definitions of shape parameters ${\hat{\eta }}_{t}^{\left(s+1\right)}$ and ${\hat{\delta }}_{t}^{\left(s+1\right)}$ are given as

(51) $${\hat{\eta }}_{t}^{\left(s+1\right)}=E^{\left(s+1\right)}\left[1-b_{t}\right]+{\hat{\eta }}_{t|t-1}$$

(52) $${\hat{\delta }}_{t}^{\left(s+1\right)}=E^{\left(s+1\right)}\left[b_{t}\right]+{\hat{\delta }}_{t|t-1}$$

**Proof:** see Appendix F.

### 3.4. Calculation of the Required Mathematical Expectations

The required mathematical expectations $E^{\left(s+1\right)}\left[\beta_{t}\right]$, $E^{\left(s+1\right)}\left[\log\left(\beta_{t}\right)\right]$,$E^{\left(s+1\right)}\left[b_{t}\right]$, $E^{\left(s+1\right)}\left[1-b_{t}\right]$, $E^{\left(s+1\right)}\left[\zeta_{t}\right]$, $E^{\left(s+1\right)}\left[1-\zeta_{t}\right]$, $E^{\left(s+1\right)}\left[\log\left(\varphi_{t}\right)\right]$, $E^{\left(s+1\right)}\left[\log\left(1-\varphi_{t}\right)\right]$, $E^{\left(s+1\right)}\left[\log\left(\gamma_{t}\right)\right],$ and $E^{\left(s+1\right)}\left[\log\left(1-\gamma_{t}\right)\right]$ in Section 3.3 are defined, respectively, as follows:

(53) $$E^{\left(s+1\right)}\left[\beta_{t}\right]=\frac{\pi_{t}^{\left(s+1\right)}}{\nu_{t}^{\left(s+1\right)}}$$

(54) $$E^{\left(s+1\right)}\left[\log\left(\beta_{t}\right)\right]=-\Psi \left(\pi_{t}^{\left(s+1\right)}\right)-\log\left(\nu_{t}^{\left(s+1\right)}\right)$$

(55) $$E^{\left(s+1\right)}\left[b_{t}\right]=\frac{p^{\left(s+1\right)}\left(b_{t}=1\right)}{p^{\left(s+1\right)}\left(b_{t}=1\right)p^{\left(s+1\right)}\left(b_{t}=0\right)}$$

(56) $$E^{\left(s+1\right)}\left[1-b_{t}\right]=1-E^{\left(s+1\right)}\left[b_{t}\right]$$

(57) $$E^{\left(s+1\right)}\left[\zeta_{t}\right]=\frac{p^{\left(s+1\right)}\left(\zeta_{t}=1\right)}{p^{\left(s+1\right)}\left(\zeta_{t}=1\right)p^{\left(s+1\right)}\left(\zeta_{t}=0\right)}$$

(58) $$E^{\left(s+1\right)}\left[1-\zeta_{t}\right]=1-E^{\left(s+1\right)}\left[\zeta_{t}\right]$$

(59) $$E^{\left(s+1\right)}\left[\log\left(\varphi_{t}\right)\right]=\Psi \left(h_{t}^{\left(s+1\right)}\right)-\Psi \left(h_{t}^{\left(s+1\right)}+d_{t}^{\left(s+1\right)}\right)$$

(60) $$E^{\left(s+1\right)}\left[\log\left(1-\varphi_{t}\right)\right]=\Psi \left(d_{t}^{\left(s+1\right)}\right)-\Psi \left(h_{t}^{\left(s+1\right)}+d_{t}^{\left(s+1\right)}\right)$$

(61) $$E^{\left(s+1\right)}\left[\log\left(\gamma_{t}\right)\right]=\Psi \left(\eta_{t}^{\left(s+1\right)}\right)-\Psi \left(\eta_{t}^{\left(s+1\right)}+\delta_{t}^{\left(s+1\right)}\right)$$

(62) $$E^{\left(s+1\right)}\left[\log\left(1-\gamma_{t}\right)\right]=\Psi \left(\delta_{t}^{\left(s+1\right)}\right)-\Psi \left(\eta_{t}^{\left(s+1\right)}+\delta_{t}^{\left(s+1\right)}\right)$$

where Ψ(·) represents the digamma function [18].

The presented variational Bayesian-based Kalman filter with UTVMLP and NSHTMN consists of Equations (18), (19) and (30)–(62). Table 1 describes the implementation of the proposed new KF.

## 4. Simulations

Aimed at demonstrating the superiority of the presented filter in the scenario with UTVMLP and NSHTMN, a numerical example is simulated. The process and measurement equations of the stochastic system are, respectively, given as [7]

(63) $$x_{t}=\left[\begin{array}{cc} 0.6 & 0.4\\ 0.1 & 0.9 \end{array}\right]x_{t-1}+e_{t-1}$$

(64) $$y_{t}=b_{t}\left[\begin{array}{cc} 1 & -2 \end{array}\right]x_{t}+g_{t}$$

where the Gaussian process noise $e_{t-1}$ and the NSHTMN $g_{t}$ are given as [12]

(65) $$e_{t-1}\sim N\left(0,Q_{t}\right)$$

(66) $$\left\{ \begin{array}{c} g_{t}\sim N\left(0,R_{t}\right)\;t\in \left[1,100\right]\left(\mathrm{Gaussian}\right)\\ g_{t}\sim \{\begin{array}{c} N\left(0,R_{t}\right)w.p.=0.98\\ N\left(0,500R_{t}\right)w.p.=0.02 \end{array}t\in \left[101,200\right]\left(\mathrm{slightly}\;\mathrm{heavy}-\mathrm{tailed}\right)\\ g_{t}\sim \{\begin{array}{c} N\left(0,R_{t}\right)w.p.=0.95\\ N\left(0,500R_{t}\right)w.p.=0.05 \end{array}t\in \left[201,300\right]\left(\mathrm{moderately}\;\mathrm{heavy}-\mathrm{tailed}\right)\\ g_{t}\sim \;N\left(0,R_{t}\right)\;t\in \left[301,400\right]\left(\mathrm{Gaussian}\right) \end{array}\right.$$

where w.p. represents “with probability”. The true process noise covariance matrix $Q_{t}$ with parameter M=1 and the nominal measurement noise covariance matrix $R_{t}$ with parameter $N=150\;m^{2}$ are set as

(67) $$Q_{t}=M\left[\begin{array}{cc} 1 & 1\\ 0 & 1 \end{array}\right]$$

(68) $$R_{t}=N$$

The real UTVMLP is set as

(69) $$p\left(\gamma_{t}\right)=\{\begin{array}{c} 0.1\;t\in \left[1,100\right]\\ \;0.15\;t\in \left[101,200\right]\\ 0.3\;t\in \left[201,300\right]\\ 0.1\;t\in \left[301,400\right] \end{array}$$

From Equations (66)–(69), it can be seen that the measurement noise and UTVMLP are divided into four stages, as shown in Table 2. The remaining system parameters are as follows: the sampling interval ∆k=0.01 s and the total simulation time T=400 s. The proposed filter is compared with the typical Kalman filter (KF) [2]; the existing variational Bayesian-based adaptive KF with UTVMLP (VBAKF) [7]; the existing Gaussian-Student’s *t*-mixture distribution-based KF (GSTKF) with Gaussian process noise [14]; and the existing IKF with known real measurement loss probability [8]. The parameters of VBAKF are selected as $p_{0}=0.5$, $\alpha_{0}=5$, $\beta_{0}=5$, ρ=1−exp(−5), and $N_{m}=10$. The parameters of GSTKF are selected as $v_{t}=5$ and $e_{0}=0.85$. The parameters of the proposed filter are given as ρ=0.99, μ=5, $h_{0}=0.85$, $N_{I}=10$, $\eta_{0}=5$, and $\delta_{t}=5$, $\varsigma =10^{-16}$. All filters are programmed with MATLAB R2018a and run on a computer with Intel Core i5-6300HQ CPU at 2.30 GHz and 8 GB of RAM.

Aimed at evaluating the performances in the estimation of the system state vector of all the algorithms, the root-mean square error (RMSE) and the averaged root-mean square error (AGRMSE) are utilized as performance indicators. The definitions of RMSE and AGRMSE of the system state are given as

(70) $${\mathrm{RMSE}}_{x}=\sqrt{\frac{1}{M_{c}}\overset{M_{r}}{\underset{r=1}{\sum }}\left({\left(x_{t}^{r}-{\hat{x}}_{t}^{r}\right)}^{2}+{\left(y_{t}^{r}-{\hat{y}}_{t}^{r}\right)}^{2}\right)}$$

(71) $${\mathrm{AGRMSE}}_{x}=\sqrt{\frac{1}{M_{c}T}\overset{T}{\underset{t=1}{\sum }}\overset{M_{r}}{\underset{r=1}{\sum }}\left({\left(x_{t}^{r}-{\hat{x}}_{t}^{r}\right)}^{2}+{\left(y_{t}^{r}-{\hat{y}}_{t}^{r}\right)}^{2}\right)}$$

where $\left(x_{t}^{r},y_{t}^{r}\right)$ and $\left({\hat{x}}_{t}^{r},{\hat{y}}_{t}^{r}\right)$ denote the actual and estimated system state at the jth Monte Carlo run and discrete-time t, respectively. $M_{c}=500$ represents the total Monte Carlo run time.

Different from the proposed algorithm and VBAKF, the KF, IKF and GSTKF do not estimate UTVMLP. Although IKF can also address the filtering problem with measurement loss, IKF is based on the assumption that the measurement loss probability is known. Therefore, only VBAKF and the proposed algorithm participate in the comparison of the UTVMLP estimation performance.

**Figure 1:**
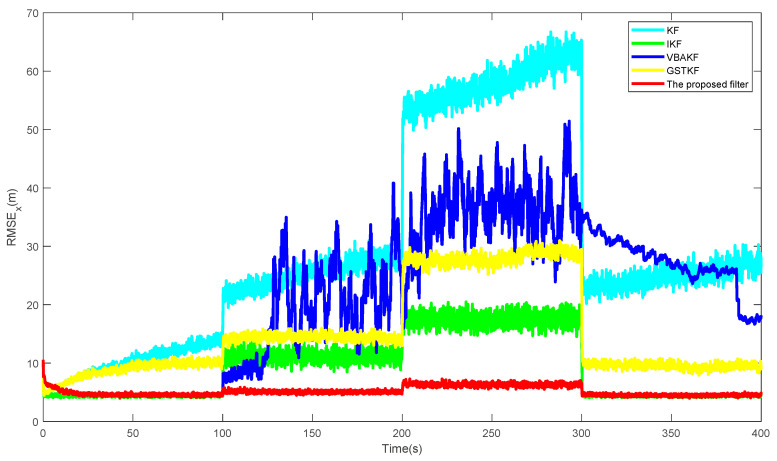
${\mathrm{RMSE}}_{x}$ of different filters.

Figure 1 shows the ${\mathrm{RMSE}}_{x}$s of the proposed filter and the existing filters over 500 times of the Monte Carlo run. Additionally, the ${\mathrm{AGRMSE}}_{x}$s and SSRTs of different filters are listed in Table 2. It can be seen from Figure 1 and Table 3 that in the contexts of UTVMLP and NSHTMN, when the measurement is the Gaussian measurement noise and there is slight loss probability, as shown in stages 1 and 4, the estimation accuracy of the proposed filter is close to the IKF with true loss probability and the performance of the proposed algorithm is better than the other algorithms. We can also find that the proposed algorithm still has better performance than the existing algorithms when the measurement has heavy-tailed measurement noise and larger measurement loss probability, as shown in stages 2 and 4. In addition, the proposed algorithm has longer SSRT and higher computational complexity than the existing filters, which can be observed from Table 3.

**Figure 2:**
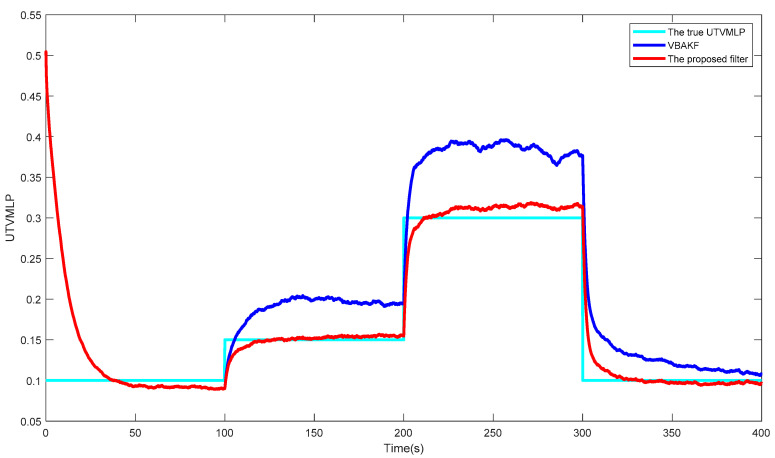
The true and estimated UTVMLPs.

Figure 2 shows the curves of the true and estimated UTVMLPs of VBAKF and the proposed filter over 500 times of the Monte Carlo run. Obviously, the NSHTMN has a great influence on the filtering performance of VBAKF and the proposed filter has better UTVMLP estimation accuracy than VBAKF in the scenario of NSHTMN.

**Figure 3:**
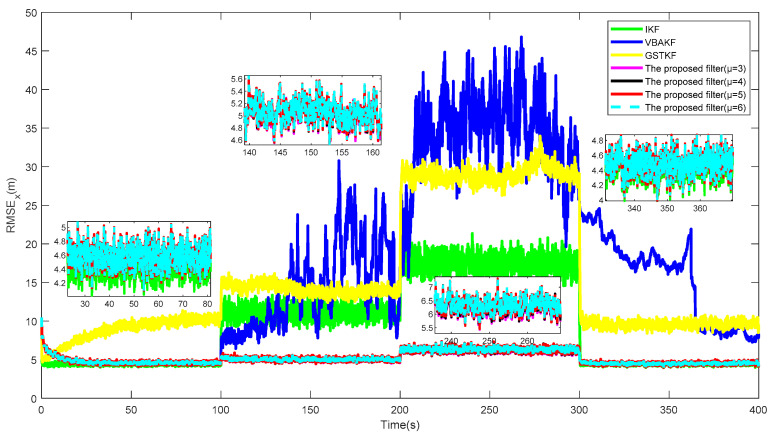
${\mathrm{RMSE}}_{x}$ of the proposed filter with μ=3,4,5,6.

**Figure 4:**
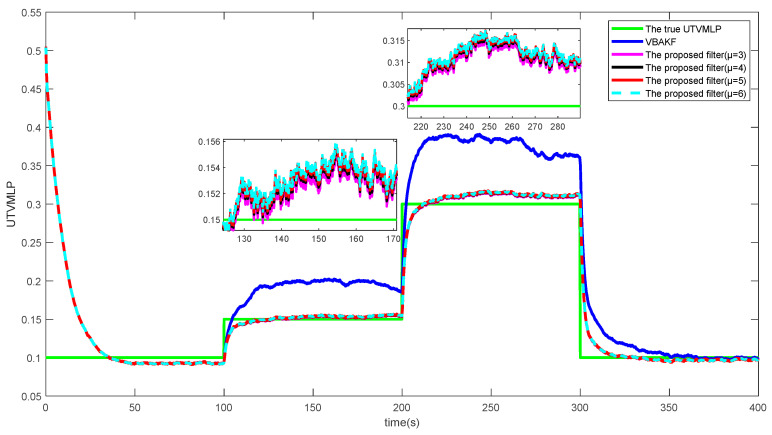
The estimated UTVMLPs of the proposed filter with μ=3,4,5,6.

Figure 3 and Figure 4 show the ${\mathrm{RMSE}}_{x}$s and the estimated UTVMLPs of the proposed filter with shape parameter μ=3,4,5,6 over the 500 Monte Carlo run, respectively. The corresponding SSRTs of the proposed filter with μ=3,4,5,6 are 0.2991, 0.2983, 0.2993, and 0.2989. It can be seen that the proposed filter with different shape parameters has better performance than current algorithms in the system state and UTVMLP estimations. Moreover, the degree of freedom parameter μ has little influence on the estimation accuracy and time complexity of the proposed algorithm, and the recommended value of μ is therefore set as 5.

**Figure 5:**
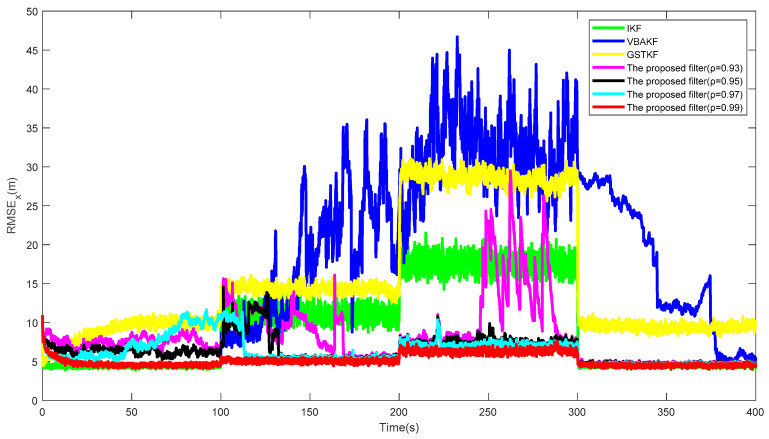
${\mathrm{RMSE}}_{x}$ of the proposed filter with forgetting factor ρ=0.93, 0.95, 0.97, and 0.99.

**Figure 6:**
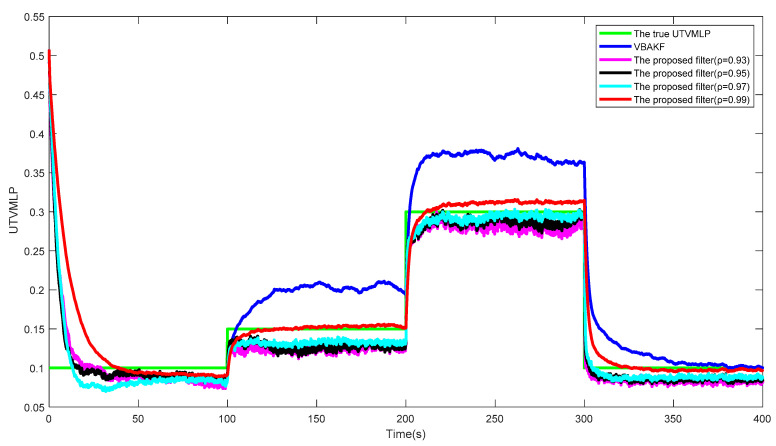
The estimated UTVMLPs of the proposed filter with ρ=0.93, 0.95, 0.97, and 0.99.

Figure 5 and Figure 6 show the ${\mathrm{RMSE}}_{x}$s and the estimated UTVMLPs of the proposed filter with forgetting factor ρ=0.93,0.95,0.97,0.99 over the 500 Monte Carlo run, respectively. The corresponding SSRT of the proposed filter with ρ=0.93,0.95,0.97,0.99 is approximately equal to 0.2990. We can find that the proposed filter with ρ=0.99 has the best performance in the system state and UTVMLP estimations, and the value of ρ has little effect on calculation complexity. Therefore, the recommended value of ρ is 0.99.

**Figure 7:**
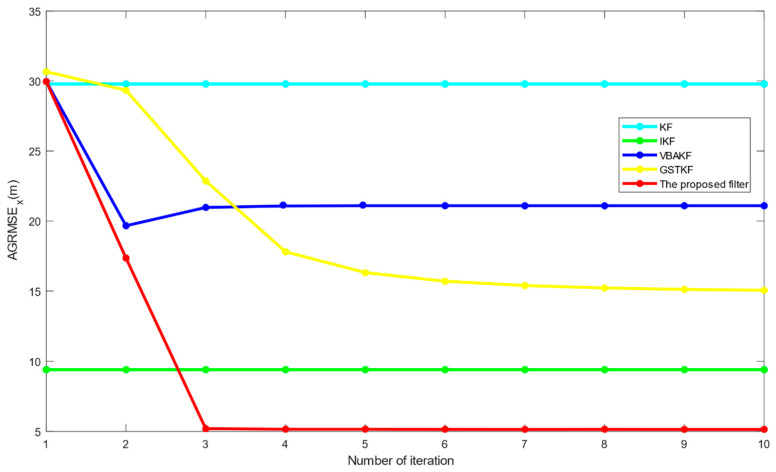
${\mathrm{AGRMSE}}_{x}$ of the proposed filter when the iteration number $N_{I}=1,2,\cdots 10$.

Figure 7 shows the ${\mathrm{AGRMSE}}_{x}$s of the proposed filter and the current algorithms with the iteration number $N_{I}=1,2,\cdots 10$. We can see from Figure 7 that the proposed filter has a smaller ${\mathrm{AGRMSE}}_{x}$ than the existing filters when $N_{I}\geq 3$ and the proposed filter converges faster than the existing filters. However, Table 4 shows that the setting of $N_{I}$ has a huge impact on the time consumption of the proposed filter and the SSRT increases with the increase of $N_{I}$. Therefore, considering time consumption and estimation accuracy, the recommended value of $N_{I}$ ranges from 4 to 10.

## 5. Conclusions

In this paper, a new VB-based KF is presented to address the filtering issue with UTVMLP and NSHTMN. The system state vector, BM, BL, the intermediate random variables, the mixing probability, and the UTVMLP are simultaneously inferred by introducing the variational Bayesian technique. Simulation results illustrated that the proposed filter has a better performance than existing filters in the estimations of the system state vector and UTVMLP.

## 6. Future Work

The environmental factors in practical applications may be more complicated than this paper illustrated. Apart from non-stationary heavy-tailed measurement noise and unknown loss probability, the system process noise may also present non-stationary heavy-tailed distribution. In terms of measurement, random delays in measurement will also appear. Therefore, in our future research, we will further consider the factors of non-stationary heavy-tailed process noise and random measurement delay based on the theoretical content of this paper. Additionally, we will design a non-linear filtering method with lower computational complexity to verify the effectiveness in the real world.

## Figures and Tables

**Table 1:** The proposed variational Bayesian-based Kalman filter with UTVMLP and NSHTMN (one-time step).

**Inputs**: ${\hat{x}}_{t-1|t-1}$, $P_{t-1|t-1}$, $Q_{t-1|t-1}$, $R_{t-1|t-1}$, $y_{t}$, $F_{t-1}$, $H_{t}$, n, m, $\mu_{t}$, $h_{0}$, ${\hat{\eta }}_{t-1}$, ${\hat{\delta }}_{t-1}$, $N_{I}$, ς
**Time update**:
1. Obtain ${\hat{x}}_{t|t-1}$ and $P_{t|t-1}$ utilizing Equations (18) and (19) (time update of typical Kalman filter).
**Variational measurement update**:
2. Initialization: ${\hat{x}}_{t|t}^{\left(0\right)}=x_{t|t-1}$, $P_{t|t}^{\left(0\right)}=P_{t|t-1}$, $E^{\left(0\right)}\left[\beta_{t}\right]=1$, $E^{\left(0\right)}\left[\log\left(\beta_{t}\right)\right]=0$, $E^{\left(0\right)}\left[b_{t}\right]={\hat{\eta }}_{t-1}/{\hat{\delta }}_{t-1}$, $E^{\left(0\right)}\left[1-b_{t}\right]=1-E^{\left(0\right)}\left[b_{t}\right]$, ${\hat{\eta }}_{t}^{\left(0\right)}={\hat{\eta }}_{t-1}$, ${\hat{\delta }}_{t}^{\left(0\right)}={\hat{\delta }}_{t-1}$, $E^{\left(0\right)}\left[\zeta_{t}\right]=1$, $E^{\left(0\right)}\left[\zeta_{t}\right]=1-E^{\left(0\right)}\left[\zeta_{t}\right]$, $E^{\left(0\right)}\left[\log\left(\varphi_{t}\right)\right]=\Psi \left(h_{0}\right)-\Psi \left(1\right)$, $E^{\left(s+1\right)}\left[\log\left(1-\varphi_{t}\right)\right]=\Psi \left(1-h_{0}\right)-\Psi \left(1\right)$, $E^{\left(s+1\right)}\left[\log\left(\gamma_{t}\right)\right]=\Psi \left({\hat{\eta }}_{t}^{\left(0\right)}\right)-\Psi \left({\hat{\eta }}_{t}^{\left(0\right)}+{\hat{\delta }}_{t}^{\left(0\right)}\right)$, $E^{\left(s+1\right)}\left[\log\left(1-\gamma_{t}\right)\right]=\Psi \left({\hat{\delta }}_{t}^{\left(0\right)}\right)-\Psi \left({\hat{\eta }}_{t}^{\left(0\right)}+{\hat{\delta }}_{t}^{\left(0\right)}\right)$
**for**$s=0:N_{I}-1$.
3. Update $q_{a}^{\left(s+1\right)}\left(x_{t}\right)$ by Equation (30).
4. Obtain ${\hat{x}}_{t|t}^{\left(s+1\right)}$, $P_{t|t}^{\left(s+1\right)}$, and ${\tilde{R}}_{t}^{\left(s+1\right)}$ by utilizing Equations (31)–(34) (typical Kalman filter).
5. Update the Gamma-distributed $q_{a}^{\left(s+1\right)}\left(\beta_{t}\right)$ by Equation (35).
6. Obtain $\pi_{t}^{\left(s+1\right)}$, $\nu_{t}^{\left(s+1\right)},$ and $G_{t}^{\left(s+1\right)}$ by utilizing Equations (36)–(38).
7. Update the Bernoulli-distributed $q_{a}^{\left(s+1\right)}\left(b_{t}\right)$.
8. Obtain $p^{\left(s+1\right)}\left(b_{t}=1\right)$, $p^{\left(s+1\right)}\left(b_{t}=0\right)$, $C_{t}^{\left(s+1\right)}$, and $D_{t}^{\left(s+1\right)}$ by utilizing Equations (39)–(42).
9. Obtain $E^{\left(s+1\right)}\left[b_{t}\right]$ and $E^{\left(s+1\right)}\left[1-b_{t}\right]$ by utilizing Equations (55) and (56).
10. Update the Bernoulli-distributed $q_{a}^{\left(s+1\right)}\left(\zeta_{t}\right)$.
11. Obtain $p^{\left(s+1\right)}\left(\zeta_{t}=1\right)$, $p^{\left(s+1\right)}\left(\zeta_{t}=0\right)$, $V_{t}^{\left(s+1\right)},$ and $W_{t}^{\left(s+1\right)}$ by utilizing Equations (43)–(46).
12. Obtain $E^{\left(s+1\right)}\left[\zeta_{t}\right]$ and $E^{\left(s+1\right)}\left[1-\zeta_{t}\right]$ by utilizing Equations (57) and (58).
13. Update the Beta-distributed $q_{a}^{\left(s+1\right)}\left(\varphi_{t}\right)$ by Equation (47).
14. Obtain $h_{t}^{\left(s+1\right)}$ and $d_{t}^{\left(s+1\right)}$ by utilizing Equations (48) and (49).
15. Obtain $E^{\left(s+1\right)}\left[\log\left(\varphi_{t}\right)\right]$ and $E^{\left(s+1\right)}\left[\log\left(1-\varphi_{t}\right)\right]$ by utilizing Equations (59) and (60).
16. Update the Beta-distributed $q_{a}^{\left(s+1\right)}\left(\gamma_{t}\right)$ by Equation (50).
17. Obtain ${\hat{\eta }}_{t}^{\left(s+1\right)}$ and ${\hat{\delta }}_{t}^{\left(s+1\right)}$ by utilizing Equations (51) and (52).
18. Obtain $E^{\left(s+1\right)}\left[\log\left(\gamma_{t}\right)\right]$ and $E^{\left(s+1\right)}\left[\log\left(1-\gamma_{t}\right)\right]$ by utilizing Equations (61) and (62).
19. If $\left(∥{\hat{x}}_{t|t}^{\left(s+1\right)}-{\hat{x}}_{t|t}^{\left(s\right)}∥/∥{\hat{x}}_{t|t}^{\left(s\right)}∥\right)\leq \varsigma$, the iteration stopped.
**End for:**
20. ${\hat{x}}_{t|t}={\hat{x}}_{t|t}^{\left(s\right)}$, $P_{t|t}=P_{t|t}^{\left(s\right)}$, $h_{t}=h_{t}^{\left(s\right)}$, $d_{t}=d_{t}^{\left(s\right)}$, $\eta_{t}=\eta_{t}^{\left(s\right)}$, $\delta_{t}=\delta_{t}^{\left(s\right)}$
**Outputs**: ${\hat{x}}_{t|t}$, $P_{t|t}$, $h_{t}$, $d_{t}$, $\eta_{t}$, $\delta_{t}$, $h_{t}/\left(h_{t}+d_{t}\right)$, $\eta_{t}/\left(\eta_{t}+\delta_{t}\right)$

**Table 2:** The stages of measurement and the corresponding measurement noise and UTVMLP.

Measurement Stage	Measurement Noise	UTVMLP
Stage 1, time 1 s~100s	$g_{t}\sim N\left(0,R_{t}\right)$ (Gaussian)	0.1 (slight loss)
Stage 2, time 101 s~200 s	$g_{t}\sim \{\begin{array}{c} N\left(0,R_{t}\right)w.p.=0.98\\ N\left(0,500R_{t}\right)w.p.=0.02 \end{array}$ (slightly heavy-tailed)	0.15 (slight loss)
Stage 3, time 201 s~300 s	$g_{t}\sim \{\begin{array}{c} N\left(0,R_{t}\right)w.p.=0.95\\ N\left(0,500R_{t}\right)w.p.=0.05 \end{array}$ (moderately heavy-tailed)	0.3 (moderate loss)
Stage 4, time 301 s~400 s	$g_{t}\sim \;\;N\left(0,R_{t}\right)$ (Gaussian)	0.1 (slight loss)

**Table 3:** ${\mathrm{AGRMSE}}_{x}$s and single-step running times (SSRT) of different filters.

Filters	KF	IKF	VBAKF	GSTKF	The Proposed Filter
${\mathrm{AGRMSE}}_{x}$ in Stage 1	10.1336	4.4326	4.7705	8.7683	4.7704
${\mathrm{AGRMSE}}_{x}$ in Stage 2	25.4009	11.1049	18.7603	14.4343	5.0660
${\mathrm{AGRMSE}}_{x}$ in Stage 3	58.0561	17.5274	35.8194	28.0354	6.3044
${\mathrm{AGRMSE}}_{x}$ in Stage 4	24.9129	4.4448	27.0252	9.5892	4.5081
${\mathrm{AGRMSE}}_{x}$ in all stages	29.6259	9.3774	21.5944	15.2068	5.1623
SSRT (ms)	0.0276	0.0925	0.1166	0.1491	0.2989

**Table 4:** SSRT of the proposed filter with the iteration number $N_{I}=1,2,\cdots 10$.

**Iteration Number** $N_{I}$	**1**	**2**	**3**	**4**	**5**
SSRT (ms)	0.0327	0.0550	0.0894	0.1307	0.1570
**Iteration Number** $N_{I}$	**6**	**7**	**8**	**9**	**10**
SSRT (ms)	0.1883	0.2243	0.2451	0.2835	0.2989

## Data Availability

No new data were created or analyzed in this study. Data sharing is not applicable to this article.

## Appendix A. Proof of Proposition 1

Utilizing Equation (29), $\log p\left(\Xi ,y_{1:t}\right)$ can be derived as

(A1) $$\begin{array}{ll} \log p\left(\Xi ,y_{1:t}\right) & =-0.5\zeta_{t}b_{t}{\left(y_{t}-H_{t}x_{t}\right)}^{T}R_{t}^{-1}\left(y_{t}-H_{t}x_{t}\right)-0.5\left(1-\zeta_{t}\right)b_{t}\beta_{t}{\left(y_{t}-H_{t}x_{t}\right)}^{T}R_{t}^{-1}\left(y_{t}-H_{t}x_{t}\right)\\ & -0.5\zeta_{t}\left(1-b_{t}\right)y_{t}^{T}R_{t}^{-1}y_{t}-0.5\left(1-\zeta_{t}\right)\left(1-b_{t}\right)\beta_{t}y_{t}^{T}R_{t}^{-1}y_{t}-0.5{\left(x_{t}-{\hat{x}}_{t|t-1}\right)}^{T}P_{t|t-1}^{-1}\\ & \times \left(x_{t}-{\hat{x}}_{t|t-1}\right)-\mathrm{logG}\left(\beta_{t},\frac{\mu_{t}}{2},\frac{\mu_{t}}{2}\right)+\zeta_{t}\log\varphi_{t}+\left(1-\varphi_{t}\right)\log\left(1-\zeta_{t}\right)+\left(1-\gamma_{t}\right)\log b_{t}\\ & +\gamma_{t}\log\left(1-b_{t}\right)+\mathrm{logBe}\left(\varphi_{t};h_{0},1-h_{0}\right)+\mathrm{logBe}\left(\gamma_{t};{\hat{\eta }}_{t|t-1},{\hat{\delta }}_{t|t-1}\right)+c_{\Xi } \end{array}$$

Exploiting $\theta =x_{t}$ in Equation (28) and utilizing (a) yields

(A2) $$\begin{array}{ll} \log q_{a}^{\left(s+1\right)}\left(x_{t}\right) & =-0.5E^{\left(s\right)}\left[\zeta_{t}\right]E^{\left(s\right)}\left[b_{t}\right]{\left(y_{t}-H_{t}x_{t}\right)}^{T}R_{t}^{-1}\left(y_{t}-H_{t}x_{t}\right)-0.5E^{\left(s\right)}\left[1-\zeta_{t}\right]E^{\left(s\right)}\left[b_{t}\right]\\ & \times E^{\left(s\right)}\left[\beta_{t}\right]{\left(y_{t}-H_{t}x_{t}\right)}^{T}R_{t}^{-1}\left(y_{t}-H_{t}x_{t}\right)-0.5{\left(x_{t}-{\hat{x}}_{t|t-1}\right)}^{T}P_{t|t-1}^{-1}\left(x_{t}-{\hat{x}}_{t|t-1}\right)+c_{x_{t}} \end{array}$$

By exploiting Equation (34) in Equation (A2), the posterior PDF $q_{a}^{\left(s+1\right)}\left(x_{t}\right)$ is defined as

(A3) $$q_{a}^{\left(s+1\right)}\left(x_{t}\right)\propto N\left(x_{t};{\hat{x}}_{t|t}^{\left(s+1\right)},P_{t|t}^{\left(s+1\right)}\right)N\left(y_{t};H_{t}x_{t},{\tilde{R}}_{t}^{\left(s+1\right)}\right)$$

Based on Equation (A3), Equation (30) can be calculated and $q_{a}^{\left(s+1\right)}\left(x_{t}\right)$ is updated by utilizing the measurement update of the traditional Kalman filter.

## Appendix B. Proof of Proposition 2

Exploiting $\theta =\beta_{t}$ in Equation (28) and utilizing Equation (A1) yields

(A4) $$\begin{array}{ll} \log q_{a}^{\left(s+1\right)}\left(\beta_{t}\right) & =0.5\left(nE^{\left(s\right)}\left[1-\zeta_{t}\right]+\mu_{t}-2\right)\log\left(\beta_{t}\right)-0.5\beta_{t}E^{\left(s\right)}\left[1-\zeta_{t}\right]E^{\left(s\right)}\left[b_{t}\right]\\ & \times \mathrm{tr}\left(A_{t}^{\left(s+1\right)}R_{t}^{-1}\right)-0.5\beta_{t}E^{\left(s\right)}\left[1-\zeta_{t}\right]E^{\left(s\right)}\left[1-b_{t}\right]\mathrm{tr}\left(B_{t}^{\left(s+1\right)}R_{t}^{-1}\right)-0.5\mu_{t}\beta_{t}+c_{\beta_{t}}\\ & =0.5\left(nE^{\left(s\right)}\left[1-\zeta_{t}\right]+\mu_{t}-2\right)\log\left(\beta_{t}\right)-0.5\beta_{t}\left[\mathrm{tr}\left(G_{t}^{\left(s+1\right)}R_{t}^{-1}\right)+\mu_{t}\right]+c_{\beta_{t}} \end{array}$$

where $A_{t}^{\left(s+1\right)}$ and $B_{t}^{\left(s+1\right)}$ are defined as

(A5) $$\begin{array}{ll} A_{t}^{\left(s+1\right)} & =E^{\left(s+1\right)}\left[\left(y_{t}-H_{t}x_{t}\right){\left(y_{t}-H_{t}x_{t}\right)}^{T}\right]\\ & =H_{t}P_{t|t}^{\left(s+1\right)}H_{t}^{T}+\left(y_{t}-H_{t}{\hat{x}}_{t|t}^{\left(s+1\right)}\right){\left(y_{t}-H_{t}{\hat{x}}_{t|t}^{\left(s+1\right)}\right)}^{T} \end{array}$$

(A6) $$B_{t}^{\left(s+1\right)}=E^{\left(s+1\right)}\left[y_{t}{\left(y_{t}\right)}^{T}\right]=y_{t}{\left(y_{t}\right)}^{T}$$

By exploiting Equations (36) and (37) in Equation (A5), we have

(A7) $$\log q_{a}^{\left(s+1\right)}\left(\beta_{t}\right)=\left(\pi_{t}^{\left(s+1\right)}-1\right)\log\left(\beta_{t}\right)-\nu_{t}^{\left(s+1\right)}\beta_{t}+c_{\beta_{t}}$$

According to Equation (A7), we can obtain

(A8) $$q_{a}^{\left(s+1\right)}\left(\beta_{t}\right)\propto \beta_{t}{}^{\left(\pi_{t}^{\left(s+1\right)}-1\right)}\exp\left(-\nu_{t}^{\left(s+1\right)}\beta_{t}\right)$$

Based on Equation (A8), Equation (41) can be obtained.

## Appendix C. Proof of Proposition 3

Exploiting $\theta =b_{t}$ in Equation (28) and utilizing Equation (A1) yields

(A9) $$\log q_{a}^{\left(s+1\right)}\left(b_{t}\right)=b_{t}\Pi_{ta}^{\left(s+1\right)}+\left(1-b_{t}\right)\Pi_{tb}^{\left(s+1\right)}+c_{b_{t}}$$

where the intermediate variables $\Pi_{ta}^{\left(s+1\right)}$ and $\Pi_{tb}^{\left(s+1\right)}$ are defined as

(A10) $$\Pi_{ta}^{\left(s+1\right)}=E^{\left(s\right)}\left[\log\left(1-\gamma_{t}\right)\right]-0.5\mathrm{tr}\left(C_{t}^{\left(s+1\right)}R_{t}^{-1}\right)+0.5nE^{\left(s\right)}\left[1-\zeta_{t}\right]E^{\left(s\right)}\left[\log\left(\beta_{t}\right)\right]$$

(A11) $$\Pi_{tb}^{\left(s+1\right)}=E^{\left(s\right)}E^{\left(s\right)}\left[\log\left(\gamma_{t}\right)\right]-0.5tr\left(D_{t}^{\left(s+1\right)}R_{t}^{-1}\right)+0.5nE^{\left(s\right)}\left[1-\zeta_{t}\right]E^{\left(s\right)}\left[\log\left(\beta_{t}\right)\right]$$

By utilizing (A9)–(A11), the posterior PMF $q_{a}^{\left(s+1\right)}\left(b_{t}\right)$ can be formulated as

(A12) $$q_{a}^{\left(s+1\right)}\left(b_{t}\right)={\left[{∆}^{\left(s+1\right)}\exp\left(\Pi_{ta}^{\left(s+1\right)}\right)\right]}^{b_{t}}{\left[{∆}^{\left(s+1\right)}\exp\left(\Pi_{tb}^{\left(s+1\right)}\right)\right]}^{\left(1-b_{t}\right)}$$

Based on Equation (A12), $q_{a}^{\left(s+1\right)}\left(b_{t}\right)$ can be updated as the Bernoulli distribution.

## Appendix D. Proof of Proposition 4

Exploiting $\theta =\zeta_{t}$ in Equation (28) and utilizing Equation (A1) yields

(A13) $$\log q_{a}^{\left(s+1\right)}\left(\zeta_{t}\right)=\zeta_{t}\Sigma_{ta}^{\left(s+1\right)}+\left(1-\zeta_{t}\right)\Sigma_{tb}^{\left(s+1\right)}+c_{b_{t}}$$

where the intermediate variables $\Sigma_{ta}^{\left(s+1\right)}$ and $\Sigma_{tb}^{\left(s+1\right)}$ are defined as

(A14) $$\Sigma_{ta}^{\left(s+1\right)}=\left\{E^{\left(s\right)}\left[\log\left(\varphi_{t}\right)\right]-0.5\mathrm{tr}\left(V_{t}^{\left(s+1\right)}R_{t}^{-1}\right)\right\}$$

(A15) $$\Sigma_{tb}^{\left(s+1\right)}=E^{\left(s\right)}\left[\log\left(1-\varphi_{t}\right)\right]-0.5\mathrm{tr}\left(W_{t}^{\left(s+1\right)}R_{t}^{-1}\right)+0.5nE^{\left(s\right)}\left[\log\left(\beta_{t}\right)\right]$$

By utilizing Equations (A13)–(A15), the posterior PMF $q_{a}^{\left(s+1\right)}\left(\zeta_{t}\right)$ can be formulated as

(A16) $$q_{a}^{\left(s+1\right)}\left(\zeta_{t}\right)={\left[\nabla^{\left(s+1\right)}\exp\left(\Sigma_{ta}^{\left(s+1\right)}\right)\right]}^{\zeta_{t}}{\left[\nabla^{\left(s+1\right)}\exp\left(\Sigma_{tb}^{\left(s+1\right)}\right)\right]}^{\left(1-\zeta_{t}\right)}$$

Based on Equation (A16), $q_{a}^{\left(s+1\right)}\left(\zeta_{t}\right)$ can be updated as the Bernoulli distribution.

## Appendix E. Proof of Proposition 5

Exploiting $\theta =\varphi_{t}$ in Equation (28) and utilizing Equation (A1) yields

(A17) $$\log q_{a}^{\left(s+1\right)}\left(\varphi_{t}\right)=\log\left(\varphi_{t}\right)\left\{h_{0}+E^{\left(s\right)}\left[\zeta_{t}\right]-1\right\}+\log\left(1-\varphi_{t}\right)\left\{E^{\left(s\right)}\left[1-\zeta_{t}\right]-h_{0}\right\}+c_{\varphi_{t}}$$

Substituting Equations (48) and (49) in Equation (A17) yields

(A18) $$\log q_{a}^{\left(s+1\right)}\left(\varphi_{t}\right)=\log\left(\varphi_{t}\right)\left(h_{t}^{\left(s+1\right)}-1\right)+\log\left(1-\varphi_{t}\right)\left(d_{t}^{\left(s+1\right)}+1\right)+c_{\varphi_{t}}$$

According to Equation (A18), we obtain

(A19) $$q_{a}^{\left(s+1\right)}\left(\varphi_{t}\right)\propto \varphi_{t}^{\left(h_{t}^{\left(s+1\right)}-1\right)}{\left(1-\varphi_{t}\right)}^{\left(d_{t}^{\left(s+1\right)}+1\right)}$$

Based on Equation (A19), Equation (47) is obtained.

## Appendix F. Proof of Proposition 5

Exploiting $\theta =\gamma_{t}$ in Equation (28) and utilizing Equation (A1) yields

(A20) $$\log q_{a}^{\left(s+1\right)}\left(\gamma_{t}\right)=\log\left(\gamma_{t}\right)\left({\hat{\eta }}_{t|t-1}-E^{\left(s+1\right)}\left[b_{t}\right]\right)+\log\left(1-\gamma_{t}\right)\left({\hat{\delta }}_{t|t-1}+E^{\left(s+1\right)}\left[1-b_{t}\right]\right)$$

Substituting Equations (51) and (52) in Equation (A20) yields

(A21) $$\log q_{a}^{\left(s+1\right)}\left(\gamma_{t}\right)=\log\left(\gamma_{t}\right)\left({\hat{\eta }}_{t}^{\left(s+1\right)}+1\right)+\log\left(1-\gamma_{t}\right)\left({\hat{\delta }}_{t}^{\left(s+1\right)}-1\right)+c_{\varphi_{t}}$$

According to Equation (A21), we obtain

(A22) $$q_{a}^{\left(s+1\right)}\left(\gamma_{t}\right)\propto \gamma_{t}^{\left({\hat{\eta }}_{t}^{\left(s+1\right)}+1\right)}{\left(1-\gamma_{t}\right)}^{\left({\hat{\delta }}_{t}^{\left(s+1\right)}-1\right)}$$

Based on Equation (A22), Equation (50) is obtained.
